# Facet-Mapping Therapy: The Potential of Facet Theory Philosophy and Declarative Mapping Sentences Within a Therapeutic Setting

**DOI:** 10.3389/fpsyg.2019.01223

**Published:** 2019-05-29

**Authors:** Paul M. W. Hackett

**Affiliations:** ^1^School of Communication, Emerson College, Boston, MA, United States; ^2^Department of Philosophy, Durham University, Durham, United Kingdom

**Keywords:** facet theory, mapping sentences, declarative mapping sentence, therapy, counseling

## Introduction: Theoretical and Philosophical Foundations

When we think about our lives we usually do not conceive an undifferentiated existential whole. Instead, we tend to divide our experiences into sub-domains or facets: our work, our family, our friends, fun activities, money earning activities, things that are meaningful to us, things that are less important and we would perhaps rather not have to do, etc. We also sub-divide these facets into elements, for example a work facet into the jobs we do, place we work, people we work with. Thus, when attempting to understand how an individual experiences his or her life, considerations must be both ontological and mereological. By ontological I am meaning that we need to adopt a perspective that embraces: (1) our understanding of our nature of being; (2) the interrelated categories and concepts that are associated to a specified domain. Ontology also involves the relationships between the components within the ontology in terms of part to whole associations, or mereology.

My approach is rooted in facet theory but does not employ all of the components of the approach [the interested reader is guided to the seminal works in facet theory development by: Guttman (1971, 1977), Borg (1977), Canter (1985), and Shye et al. (1994)]. I have claimed elsewhere that facet theory when used with a declarative mapping sentence is concerned with both the ontological and mereological understanding of a research domain (Hackett, 2014, 2016, 2018a,b, 2019; Greggor and Hackett, 2018). A researcher who is adopting facet theory will conceive the research they are conducting as possessing multiple variables (both experimental and outcome) that will in some interactive sense be significant within the domain of their enquiry.

## Qualitative and Quantitative Experiences

Consideration of the above listed sub-domains (facets) suggests that these are not all identified using the same criteria. For example, we may expect the facets of work, friends, and family to usually be qualitatively differentiable. Therefore, these facets are unlikely to be understood in terms of their being better or worse, more or less, greater or lesser, etc. Rather, they are likely to be simply distinct or qualitatively different from each other. Thus, family experiences may be seen as distinct from work experiences but not necessarily as better or worse, more or less, etc. In order to differentiate such experiences in terms of extent they may have to be reference to another, more quantitative facets. For example quantitative facets may include any sub-domain that can be thought of in terms of better or worse, more or less, greater or lesser, etc., and these could include, fun activities, money earning activities, things that are meaningful to us, things that we do not enjoy, etc. On this understanding the facet of family (for example) could be evaluated as more or less in terms as it is related to other facets such as fun activities. It is therefore through the combination of the two facets, one qualitative, and a second quantitative facet, that a particular activity, state of being or event that involves a person's family can be understood as it impacts on and is experienced by an individual. Finally, there are facets of our lives that place experiential facets into a context, which is either internal or external to the individual. Examples of these would be places, individual health, times, etc.

## Using Facet-Mapping Therapeutically

Above, I have suggested how life experiences can be conceived as being ontological and mereological. I have also suggested that breaking life into facets may assist in our understanding the qualitative and quantitative variables (life events) that form our life experiences. On this conception, the role of the therapist can be conceived of as being that of a person who explores a client's life with them and facet-mapping is firmly rooted in what Sklare (2014) calls the assumption that in therapy the client knows him or herself best. The therapist and client together identify facets of the client's life and attempt to consider the interplay between facets, or, as Martin (2015) puts this, “I (the therapist) am a fellow explorer going through this with you, but you are the final authority.” Understanding facets of a person's life, how these interrelate to each other and what the outcome of specific levels of each facet when these interrelate with other levels of other facets, can assist the therapist in forming a picture of the client's experiential world and how this relates to discomfort or pathological issues. I have found it useful when working with a client to take time to identify the facets in a person's life that they see as being important in terms of a problem or difficulty they are experiencing and how facets are expressed. Having assembled a list of facets and their elements these may be joined together during discussion with the client in the form of a declarative mapping sentence.

It is important to note that the construction of a declarative mapping sentence does not have to be necessarily co-constructive. Rather, it is the position that is assumed by the therapist that enables co-construction of a mapping. Moreover, a counseling process may be client-centered without the employment of a mapping sentence. However, the advantage that I have found in co-constructing a declarative mapping sentence with a client is that it actively involves both client and therapist, both verbally and physically (through writing the sentence), in an exploration which requires thought and reflection by both parts of this dyad.

In a therapeutic setting the therapist may try to be comprehensive by attempting to clearly locate an issue in terms of a client's whole life. Attempting to focus upon the particular issues a client has impels the therapist to concentrate upon the specific aspects of a client's life that are of concern to the client. If a therapist takes too broad an outlook this may make the therapy too general to have a significant effect. Conversely, by embracing too narrow a view may miss other significant factors associated with the client's issue(s). Adopting a facet-mapping approach and assembling a declarative mapping sentence of the problem area requires the client and therapist to identify a discrete set of issues and their influences within the client's experiences.

In the research literature facets are typically of a fixed number of structures and these facets have been discovered to interact in specific ways (see, for example, Levy, 1985). As well as being quantitative or quantitative facets may also be focusing facets or layering facets. Facets may come together in a number of ways, for example as a cylindrex, which is a structure that has been found in research into attitudes, values, etc. A cylindrex may be imagined as a layer cake with multiple layers. In this cake, each layer is more similar to adjacent layers than more distant layers, where the top and bottom layers are the most dissimilar. We may also imagine that in the center of the cake the flavor is very intense and that this intensity decreases as we progress toward the outside edges of the cake with there being little flavor at the outside edge. Finally, the cake may be sliced into wedges originating from the center where each slice has a different color. Therapeutically, the illustration of a cake may be discussed and the layers, rings, and wedges identified. Indeed, the aim of therapy is for the client and therapist to “get to know” the structure of the cake. It is also possible for clients to identify where they are positioned in the cake in relation to different circumstances. As has been noted (e.g., Morgan, 2000; Andrews, 2017), narrative statements can be powerful in therapy. It is my opinion that declarative mapping sentences have the ability to personalize such statements and to target therapy.

An example may help to illustrate my claim. If we imagine that a client has a problem at work a declarative mapping sentence may be assembled that identified the major facets as being, job task, degree of personal skill involved, and physical location and let us imagine that these are represented by wedges in the cake. The levels of skill required of our client are represented by the concentric circles emanating from the center of the cake (with more personal skill tasks being positioned centrally and the peripheral items being those that require less skill). The different locations where the client works are the layers of the cake. Situations at work may be located within the cake and situations in which the client is experiencing difficulty may be explored through reference to the cakes structure and the interaction of the facets and elements that together form the cake. Different tasks and events at work may be compared in terms of their relative location in the cake. I have found it useful to use a wooden model of a cylindrex and to let the client handle this when they are discussing their issues. This may reveal surprising influences of a facet in reference to an event of which the client was not previously aware. In this way, the model or a drawn representation of the model may be used in counseling with a client. Alice Morgan makes a similar point when she speaks about what White (1992) calls the statement of a position map. She sees this as involving the negotiating and naming of a client's issue in a way that is congruent with that person's meaning and experience of the problem, exploring the impact of the problem in the person's life, asking the client to evaluate the impact of the issues identified and asking them to justify their claims.

The facet-mapping approach can allow the identification of facets, their elements, the roles of facets, the importance/salience of facets, how facets interact, how working on one facet of life can influence another or other facets and the relative importance of facets in terms of the client's issues. After a client has discussed their mapping sentence and how the sentence may be depicted in a three-dimensional representation, the therapist may suggest how the facets can be re-assembled through the client changing their actions, thoughts, etc., and the outcome of such changes discussed in reference to the model.

A simplified example of how I used the approach with a client is provided in the case of “Terry.” I was counseling Terry because of his problems in the life areas of substance abuse, his unemployment, and issues he had with his partner. Terry and I initially identified these life areas in the development of a declarative mapping sentence. Elements were identified of what were for Terry relatively discrete aspects of each of these life areas. For example, when talking about his partner it emerged that Terry identified her as providing him with his main source of income, as being a source of constraint upon his life-style and also as providing him with emotional support. When speaking about the other two life areas (unemployment and substance abuse) elements emerged that were in some ways both similar and different to those of the partner facet (for example, the elements of having time to do things, affording to go out with his mates, getting money from the government were identified for the unemployment facet). Terry initially positioned the facets in the following order: partner; employment; substance abuse. Having identified the facets and their elements in the declarative mapping sentence we then used the wooden cylindrex model and identified the facets as the circular disks with the elements as wedges in these disks. Issues were also seen to occupy elements in which issues positioned nearer the center of each disk were identified as being of greater importance to Terry.

Terry had several “ah ha” moments when we used this model. For example, he discovered that the facet of partner was intimately related to the facets of unemployment and substance abuse and was better positioned between these two facets. Furthermore, it became apparent that what he had initially identified as being a discrete and relatively important part of his life, the income he received from his partner, was in fact central in this model and indeed that it was not really a discrete facet but rather ran through many of the other facets and elements. Having identified these changes in the model, I was able to talk with Terry about how this related to the violence he had displayed toward his partner and the resentment he felt toward her for paying for his life-style. We were also able to suggest how by changing his thinking about his partner's financial support from existing on its own as a separate facet and moving this conception to being intertwined amongst other facets could suggest ways he may change his behavior toward her. I will not go further into this case as I provide it as an illustration of how first identifying the pertinent facets and elements in a declarative mapping sentence and then exploring the interrelationship of facets and elements in a model was able to facilitate issue recognition and suggest behavior change.

## Conclusions and the Future

By exploring a person's life using the facet-mapping approach the client and therapist assume the roles of co-explorers where such roles may assist in the important task of dispelling notions that the client is in someway crazy but rather that they are rational joint “explorateurs.” Future research could enquire into the veracity of this claim. In the future, there is a need for software to be developed that enables a touch-operated model to be built on a tablet. This would allow the therapist and client to travel together more easily through a life domain, issue area, etc., in a way that is perhaps more user-friendly than the wooden model that I have used. Further research is also needed to enquire into the limitations of the facet mapping approach, in terms of the therapeutic situations in which this is a more or less helpful approach to adopt. It has been my experience when developing a mapping sentence in a counseling situation that the process of declarative mapping sentence development can be initially completed in a single session and then refined and modified in later sessions. However, the optimal time this process should take requires further investigation. Eighty years of using facet theory within social science research has demonstrated that the mapping sentence is a flexibility structure that is able to capture and allow the exploration of complex human experiences. Furthermore, the employment of facet theoretical approaches in research design have suggested that when a research question is stated in terms of its facets, that this question usually possesses two to four facets. To date, this has also been my experience when using a declarative mapping sentence where I have found that ~3 facets usually emerge during the development of the sentence. However, further research is needed to determine the flexibility of the declarative mapping sentence and the optimal number of facets that should be present (if such an optimal number does indeed exist) to facilitate a client and therapist co-exploration of complex therapeutic issues.

## Author Contributions

The author confirms being the sole contributor of this work and has approved it for publication.

### Conflict of Interest Statement

The author declares that the research was conducted in the absence of any commercial or financial relationships that could be construed as a potential conflict of interest.

## References

[B1] Andrews B. (2017). Cognitive Behavioral Therapy (CBT): Master Your Brain and Emotions to Overcome Anxiety, Depression and Negative Thoughts (CBT Self Help Book 1- Cognitive Behavioral Therapy). Scotts Valley, CA: CreateSpace Independent Publishing Platform.

[B2] Borg I. (1977). Some basic concepts of facet theory, in Geometric Representations of Relational Data, ed Lingoes J. (Ann Arbor, MI: Mathesis Press), 65–102.

[B3] Canter D. (ed.). (1985). Facet Theory: Approaches to Social Research. New York, NY: Springer Verlag.

[B4] Greggor A. L. Hackett P. M. W. (2018). Categorization by the animal mind, in Mereologies, Ontologies and Facets: The Categorial Structure of Reality, ed Hackett P. M. W. (Lanham, MD: Lexington Books), 1–18.

[B5] Guttman L. (1971). Measurement as structural theory. Psychometrika 36, 329–347.

[B6] Guttman L. (1977). What is Not What in Statistics. Statistician 26, 81–107.

[B7] Hackett P. M. W. (2014). Facet Theory and the Mapping Sentence: Evolving Philosophy, Use and Application. Basingstoke: Palgrave.

[B8] Hackett P. M. W. (2016). Facet theory and the mapping sentence as hermeneutically consistent structured meta-ontology and structured meta-mereology. Front. Psychol. 7:471. 10.3389/fpsyg.2016.00471 27065932 PMC4814565

[B9] Hackett P. M. W. (2018b). Declarative mapping sentence mereologies: categories from aristotle to lowe, in Mereologies, Ontologies and Facets: The Categorial Structure of Reality, ed Hackett P. M. W. (Lanham, MD: Lexington Publishers), 135–160.

[B10] Hackett P. M. W. (2019). Declarative Mapping Sentences as a Co-ordinating Framework for Qualitative Health and Wellbeing Research. J. Soc. Sci. Allied Health Profes. 2, E1–E6.

[B11] Hackett P. M. W. (ed.). (2018a). Mereologies, Ontologies and Facets: The Categorial Structure of Reality. Lanham, MD: Lexington Books.

[B12] Levy S. (1985). Lawful roles of facets in social theories, in Facet Theory: Approaches to Social Research, ed Canter D. (New York, NY: Springer Verlag), 59–96.

[B13] Martin D. G. (2015). Counseling and Therapy Skills. Long Grove, Il: Waveland Press Inc.

[B14] Morgan A. (2000). What is Narrative Therapy?: An Easy to Read Introduction. Adelaide, SA: Dulwich Centre Publications.

[B15] Shye S. Elizur D. Hoffman M. (1994). Introduction to Facet Theory: Content Design and Intrinsic Data Analysis in Behavioural Research. Thousand Oaks, CA: Sage Publishers.

[B16] Sklare G. B. (2014). Brief Counseling That Works: A Solution-Focused Therapy Approach for School Counselors and Other Mental Health Professionals. Thousand Oaks, CA: Corwin Press Inc.

[B17] White M. (1992). Deconstruction and therapy, in Experience, Contradiction, Narrative and Imagination, eds Epstone D. White M. (Adelaide, SA: Dulwich Centre Publications). 3:21–40

